# Pediatric Granular Cell Tumor of the Breast: A Case Report and Review of the Literature

**DOI:** 10.1155/2015/568940

**Published:** 2015-09-27

**Authors:** Nathan P. Heinzerling, Shannon M. Koehler, Sara Szabo, Amy J. Wagner

**Affiliations:** ^1^Division of Pediatric Surgery, Children's Hospital of Wisconsin, 8701 Watertown Plank Road, Milwaukee, WI 53226, USA; ^2^Division of Pathology, Children's Hospital of Wisconsin, 8701 Watertown Plank Road, Milwaukee, WI 53226, USA

## Abstract

*Objective*. Granular cell tumors arise from neurogenic mesenchymal stem cells and can occur anywhere throughout the body. They rarely present as breast masses and should be included in the differential diagnosis of pediatric breast neoplasms. We report a rare presentation of a pediatric breast granular cell tumor and a review of the literature. *Participant*. A 15-year-old female presented with an enlarging breast mass. She underwent ultrasound imaging and excisional biopsy, which revealed a granular cell tumor. Granular cell tumors of the breast are difficult to diagnose using ultrasound and mammography due to numerous similarities to other breast masses. Histopathologic staining best differentiates breast granular cell tumors from other breast masses with their positive staining for S100, CD68, and neurospecific enolase. *Conclusion*. Although rare, granular cell tumors of the breast should be considered as a possible diagnosis for pediatric breast masses to allow for proper management and follow-up for these patients. Although rare, these tumors do have malignant potential necessitating a correct and timely diagnosis.

## 1. Introduction

Breast disease is uncommon in pediatric patients and most cases are benign in nature. The most frequent breast tumors in adolescents are single or multiple fibroadenomas. Fortunately, breast carcinoma is extremely rare in young females with an estimated incidence of 3.2 per million women less than 25 years of age [1]. Only 0.02% of surgically removed breast masses in girls are carcinoma [2]. Although rare, granular cell tumors (GCT) of the breast should be included in the differential diagnosis for a pediatric breast mass. Granular cell tumors arise from anywhere in the body and are most commonly found in the tongue, head, and neck. The occurrence of breast GCT accounts for 5% to 15% of all GCT and 1 in 1000 of all breast tumors in a screened population [3–5].

Granular cell tumors were first described by Abrikossoff et al. in 1926 as a tumor of the tongue but were later described in the breast in 1931 [6]. He originally described the tumors as having a skeletal muscle origin due to the cytologic similarity to myogenic cells. Further descriptions included suggestions of fibroblast, histiocytic, and undifferentiated mesenchymal cell origin. However, the current consensus describes GCT as mesenchymal cells with a neurogenic origin [4, 7].

## 2. Case Presentation

**Figure 1:**
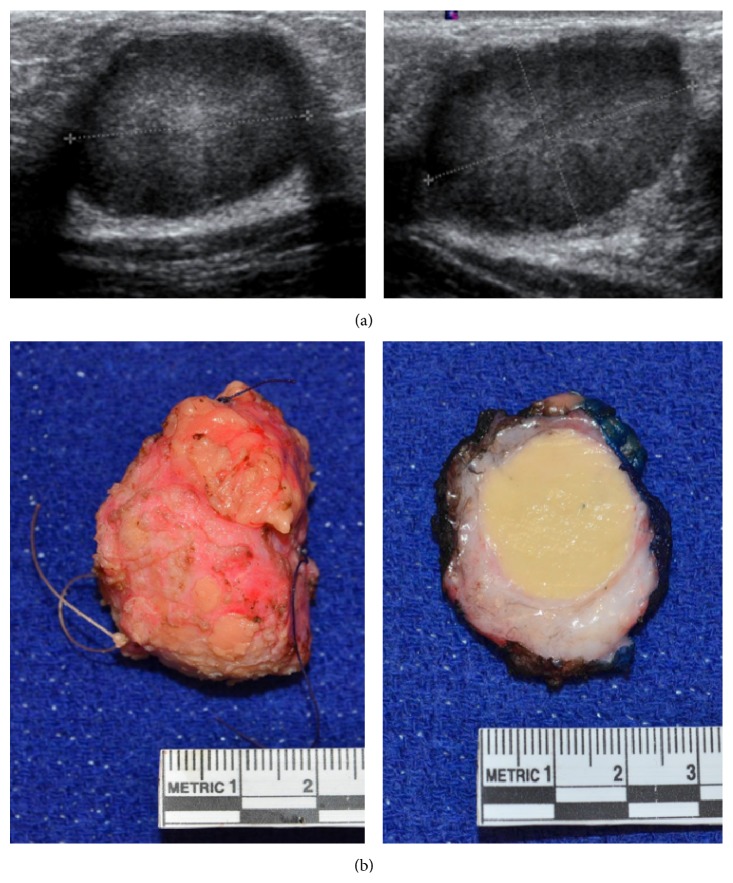
(a) Ultrasound images of a granular cell tumor of the breast showing a solitary mass without vascularity. (b) Gross pathologic images of the resected granular cell breast tumor.

An otherwise healthy, 15-year-old female was referred for evaluation of a right breast mass, identified a few months before by self-examination. She reported no associated pain, nipple discharge, or skin changes and had no family history of breast or ovarian cancer. Physical examination identified a firm, mobile mass in the right upper outer quadrant with no associated lymphadenopathy. On ultrasound examination, a solitary, solid 3 × 2.1 × 2.2-centimeter mass was noted at the 10 o'clock position, with no vascularity on Doppler exam, (Figure 1(a)) and interpreted as most consistent with a fibroadenoma.

**Figure 2:**
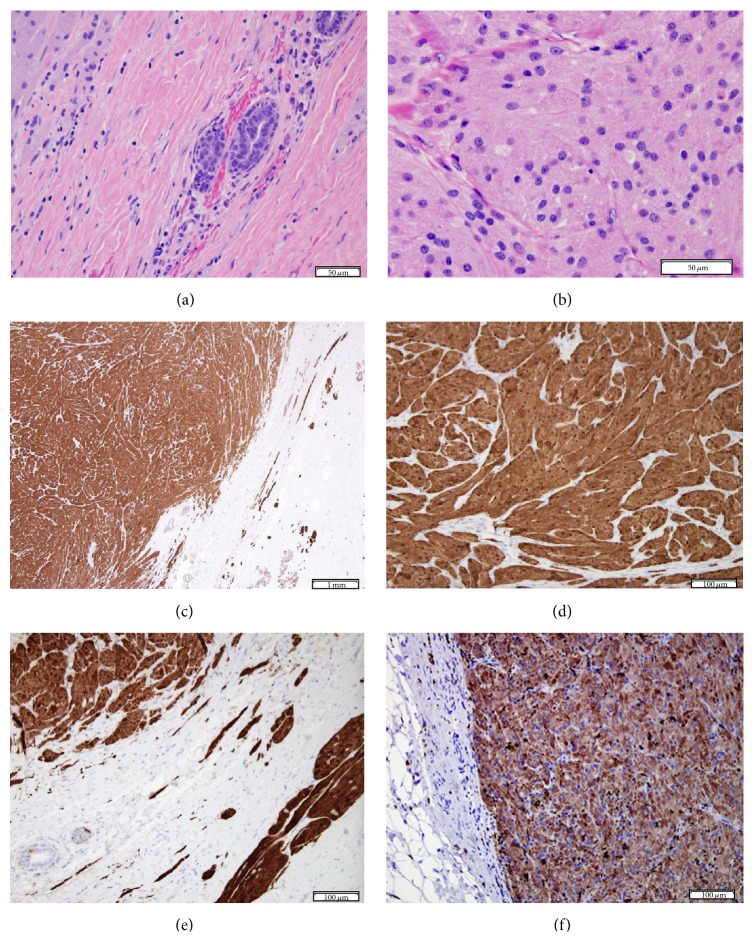
(a) Margin of tumor, cords and files of tumor cells in compressed collagenous stroma, with normal ducts, minimal inflammatory reaction (H&E). (b) Center of the tumor; granularity of the abundant eosinophilic cytoplasm is apparent (H&E). (c) Low power architecture of tumor, with swirling cords; peripherally entrapped normal duct (middle of picture); compressed, minimally infiltrative strands on the right, along with normal breast lobules Immunostain for S100. (d) Center of the tumor, positive for S100 immunohistochemical stain. (e) Margin of the tumor (Immunostain for S100); short infiltrative tumor strands (right lower quadrant of image) in compressed stroma (right upper quadrant), entrapping normal ducts (left lower corner) by the tumor margin (left upper quadrant). (f) Diffuse immunohistochemical staining of the tumor with CD68.

The patient underwent excisional biopsy of the mass through a circumareolar incision adjacent to the mass. She was discharged home on the same day and had an uncomplicated recovery. Gross pathologic exam of the 3.5 cm specimen revealed a round 2.1-centimeter, demarcated, distinct, homogenously yellow mass (Figure 1(b)). Microscopically the lesional cells were large, round to polygonal, with large amounts of granular eosinophilic cytoplasm, small nuclei, and indistinct nucleoli, and were arranged in a thick cord-like pattern with delicate vascularity. The tumor was homogenous with no cytologic atypia, notable mitoses, or necrosis. Immunohistochemical staining for CD68 and S100 was diffusely positive (Figure 2) but negative for cytokeratin, consistent with a GCT. The tumor borders were pushing and compressing the stroma at low power, with short peripheral extensions and limited peripheral encasement of occasional normal ducts and lobules at high power (Figure 2(e)).

## 3. Characteristics

Granular cell tumors typically present as slow growing, smooth, solitary nodules in the subcutaneous, intradermal, or submucosal tissue. Although described in most regions of the body, GCT are most common in the head and neck, chest wall, and arms [8]. Granular cell tumors of the breast account for 5% of all GCT cases and 0.1% of all breast tumors [6, 8]. They are most commonly diagnosed in perimenopausal women but there have been documented cases in pediatric patients [5]. Although largely a disease of females, 6.6% of breast GCT occur in men. Granular cell tumors of the breast arise from the intralobar breast stroma and occur in the distribution of the cutaneous branch of the supraclavicular nerve in the upper outer quadrant of the breast [7].

The majority of breast GCT are benign, but up to 1-2% are malignant at the time of presentation [4]. Clinically, features associated with malignancy include large mass, rapid growth, lymphadenopathy, poor circumscription, and aggressive local invasion. Histologically suggestive features, as outlined by Wang et al. [9], are necrosis, spindle-type cells, vesicular nuclei, large nucleoli, greater than five mitoses per high-powered field, high cytoplasm to nuclear ratio, and nuclear pleomorphism. With two of these six criteria they define such a mass as “atypical” and with three or more criteria as “malignant.” The few documented cases of malignant granular cell tumors of the breast have metastasized to the lymph nodes, lungs, liver, and bone [10, 11].

## 4. Imaging

Imaging techniques for the identification and description of breast masses widely employ the use of mammography. However, in young females the increased density of the breast can obscure many findings on mammography making ultrasound the modality of choice. Granular cell tumors of the breast are difficult to differentiate on both ultrasound and mammography due to numerous similarities to breast malignancies including irregularity, spiculation, stellation, isodensity, and heterogeneity [4]. The utility of MRI and PET-CT to identify GCT of the breast has yet to be determined but is still being evaluated.

## 5. Ultrasound

Ultrasound is the most common imaging modality used to evaluate breast masses in the pediatric population. In a retrospective review of female pediatric patients with breast masses evaluated by ultrasound, Sanchez et al. found that 91% of the pathologically examined masses were fibroadenomas [12]. While ultrasound is an effective tool for identifying fibroadenomas, it remains difficult to reliably differentiate GCT of the breast from other tumors, as the features are rather nonspecific and some may mimic malignancy. The range of ultrasound features of breast GCT is broad and includes a poorly defined solid mass, a high depth to width ratio, generally hypoechoic with posterior shadowing and heterogeneity with coarse internal echo. Peripheral hypervascular echo texture is an inconsistent finding [3, 13]. While ultrasound is a useful tool to identify granular cell tumors, it is unable to differentiate it from other breast neoplasms.

## 6. Mammography

As noted above, mammography is a largely ineffective modality for imaging breast masses in pediatric patients due to the increased density of the breast. Also, the mammographic findings of GCT are highly variable from benign appearing nodules to spiculated masses with associated skin changes highly suspicious for malignancy. Mammographic features of GCT of the breast may include irregularity, spiculation, isodensity, stellation, and variable tendril-like extensions [4]. In addition to these findings, the mass may have associated skin thickening and skin dimpling due to a severe desmoplastic reaction in the surrounding tissue. Of note, calcifications are not seen in GCT of the breast and when found should raise concern for malignancy. Adeniran et al. and Irshad et al. include numerous mammographic images of GCT and can be referenced for comparison [3, 7].

## 7. Fine Needle Aspiration

Preoperative biopsy can be helpful in managing breast masses and allows for patient counseling and operative planning. Fine needle aspiration is a safe option for obtaining tissue preoperatively. Miller et al. reported a sensitivity of 68 to 93% for fine needle aspiration in the diagnosis of GCT of the breast [13]. The accuracy was increased with the utilization of ultrasound to allow for guided biopsies. However, cytologic examination of fine needle aspiration samples is associated with numerous pitfalls including the solidity of the mass, alteration of the cellular architecture, and insufficient material for immunohistochemical staining. Therefore, fine needle aspiration is not generally recommended for evaluating breast masses.

## 8. Core Needle and Excisional Biopsy

The use of an 11- to 18-gauge needle to obtain a core needle biopsy improves on fine needle aspiration by preserving the cytologic architecture of the specimen and obtaining enough tissue to allow for immunohistochemical staining. In Brown et al.'s study of a case cohort of breast GCT, all masses examined preoperatively with a core biopsy were correctly diagnosed [4]. However, excisional biopsy remains the gold standard for definitive diagnosis and treatment of a GCT mass. With wide local excision the recurrence rate of GCT is low (2–8%) [14].

## 9. Histogenesis

The histogenesis of GCT remains controversial. The current literature describes GCT to have a Schwann cell origin using immunohistochemical markers. Initial immunohistochemical studies showed that GCT do not stain positive for alpha-tubulin or keratin, effectively ruling out a muscle or epithelial origin. Granular cell tumors stain positive for S100, a protein found in neural cells, Schwann cells, and melanocytes. Although S100 is a sensitive marker for GCT of the breast, it is not specific, as 10% of breast malignancies also stain positive for S100. Additional markers to identify granular cell tumors include CD68 and neurospecific enolase. CD68 is a marker of lysosomal activity and is found in both perineural Schwann cells and 90% of granular cell tumors [4]. Additionally, GCT gain their appearance due to the abundance of lysosomes that stain positive for CD68. Neurospecific enolase is found in neural cells and cells with neuroendocrine differentiation. These findings support the theory that GCT originate from the Schwann cells of distal nerves ending in the breast tissue.

## 10. Macroscopic Appearance

Granular cell tumors of the breast are typically solid, firm, homogenous masses with a white to tan color and typically lack areas of necrosis. The mass is nonencapsulated and known for infiltration into the surrounding breast tissue and pectoral tissue. It is the infiltrative feature that gives the impression of malignancy on imaging. Kragel et al. described 58 cases of GCT of the breast and noted that 52% of the masses were well circumscribed and 33% poorly circumscribed highlighting the heterogeneity of GCT presentation [15].

## 11. Microscopic Appearance

Granular cell tumors of the breast display large nests or sheets of uniform, polygonal cells set in a background of fibrous, hyaline stroma. These cells typically have an abundance of eosinophilic cytoplasm densely filled with lysosomes giving the cells their granular appearance. The nuclei tend to be small and round with one of two nucleoli [16]. They do not display mitoses, pleomorphism, nuclear multiplicity, or atypia. When these findings are present, concern for malignant degeneration of the tumor should be raised, as discussed earlier. The cells stain positive for periodic acid-Schiff stain and the Diff-Quik stain produces a blue color [4, 17].

## 12. Treatment

The treatment of GCT of the breast is wide local excision. Due to the benign nature of the majority of these tumors, lymph node dissections, sentinel lymph node biopsies, and mastectomy are not indicated on the initial resection. The infiltrative nature of the tumor often leads to positive margins after initial excision and should be considered for reexcision of the margin in question and requires careful long-term follow-up. As the recurrence rate is 2–8%, patients with benign GCT of the breast should be followed up annually with clinical examination to monitor for recurrence, but the utility of further imaging is unclear [17]. In cases where malignant degeneration is found, the patient should be treated with wide local excision and sentinel lymph node biopsy [18]. Unfortunately, chemotherapy and radiotherapy have not been shown to significantly improve the clinical course of the disease [9]. One study showed no recurrence in seven cases of GCT of the breast with negative margins over a median follow-up of 8 years. Additionally, the two patients with positive margins had no evidence of tumor recurrence after a median follow-up of 7.5 years despite no further interventions [19]. No follow-up was recommended in the study patient due to her negative margins and a benign tumor. Further studies would be required to determine recommended duration and frequency of follow-up of patients with positive margins or a malignant tumor.

## 13. Conclusion

Granular cell tumors of the breast are rare in both the adult and pediatric population and account for less than 0.1% of all breast tumors [3]. These tumors are thought to arise from Schwann cells in the lobular breast stroma and are most common in the upper, outer quadrant. The vast majority of GCT of the breast are benign in nature but their diagnostic difficulty lies with their ability to mimic malignant breast disease on clinical and radiographic workup. Granular cell tumors are an important part of the differential diagnosis of an adolescent breast mass as the imaging can be misleading and concerning for a malignant process that could lead to unnecessary preoperative testing and patient anxiety. Although the tumor can be locally invasive, it rarely is associated with metastasis to lymph nodes or other organs. In the pediatric population, the best mode of imaging is ultrasound, but this is largely ineffective in differentiating it from other pathologies. Diagnosis is typically made with immunohistochemistry of excisional or percutaneous biopsies that stain positive for S100 and CD68.

It is important to include GCT in the differential diagnosis on a pediatric breast mass, especially those that occur in the upper outer quadrant of the breast. Overall, GCT of the breast are typically benign in nature and have an excellent long-term prognosis with wide local excision.
